# Comparing the Fracture Resistance of Dentine Posts and Glass Fiber Posts in Primary Maxillary Incisors: An In Vitro Study

**DOI:** 10.7759/cureus.34591

**Published:** 2023-02-03

**Authors:** Ayham Hijaz, Mohamed K Altinawi, Hasan Alzoubi

**Affiliations:** 1 Department of Pediatric Dentistry, Damascus University, Damascus, SYR

**Keywords:** primary maxillary incisors, testometric, primary anterior teeth, fracture resistance, dentin posts, fiber posts

## Abstract

Background/purpose

An ideal post material should have physical and mechanical properties similar to dentin. Another problem when restoring primary teeth that have undergone root canal treatment is the availability of materials that are resorbed in the exfoliation process in a manner similar to the structure of the natural tooth, allowing the normal eruption of a permanent tooth. This study aimed to evaluate the effect of using dentine posts on the fracture resistance of endodontically treated primary incisors in comparison to glass fiber posts.

Materials and methods

The study sample consisted of 30 extracted primary maxillary incisors that were randomly distributed into the following two groups: group I (experimental, n=15), which was restored with dentine posts; and group II (control, n=15), which was restored with glass fiber posts. Initially, 10 extracted single-root permanent teeth were collected to prepare 20 dentine posts using a computer-aided design-computer-aided manufacturing (CAD-CAM) machine. Then, the crowns of maxillary primary incisors were cut and the canals were prepared and filled. Then the preparation for a post was made using Gates Glidden drills, and the posts were placed with an extension of 3 mm within the canal in both groups, then the crown was built and the teeth were placed within acrylic cubes and subjected to 500 thermocycling. Fracture resistance was recorded using a Testometric machine (Rochdale, England: Testometric Co. Ltd.). Data were analyzed using an independent Student’s t-test.

Results

The dentine posts group showed greater fracture resistance (246.3 N) than the glass fiber posts group (206.3 N). A statistically significant difference (p=0.004) was found between the two groups in favor of the dentine posts group.

Conclusion

Based on this in vitro study, the dentin posts used in restoring severely decayed primary maxillary incisors showed greater fracture resistance than glass fiber posts. Therefore, the use of dentin posts as intra-canal stabilizers in maxillary primary incisors is a good alternative for glass fiber posts.

## Introduction

Dental caries is the most common chronic disease in childhood [1]. These caries show a distinctive clinical pattern and are located mainly on the maxillary primary incisors in addition to the first primary molars, while the primary canines are less affected and usually the mandibular primary incisors are unaffected [2]. Severe caries of the maxillary primary incisors usually leads to pulp damage and in some cases, a loss of the entire coronal structure can occur. Hence, extraction was the only treatment option previously, which resulted in several problems, such as speech problems, chewing disorders, and the child’s psychological problems [3].

Conservative restorative treatments for primary anterior teeth have always represented a major challenge in pediatric dentistry due to the small size of the crowns and the expansion of the size of the pulp chamber, without forgetting the esthetic factor, which is an important consideration in this area, which means that there is an indication for composite restorations [4]. Additional difficulties arise due to the structural differences between permanent and primary teeth, which do not provide sufficient bonding forces for composite restorations due to the lack of space for the dental tissues available for bonding [5].

For severely decayed incisors, endodontic treatment is a required procedure, and then the use of intra-canal stabilizers is necessary to provide support and durability for coronal restoration [6]. In this context, there are many options available, such as orthodontic "α" wire posts [7], orthodontic "γ" wire posts (γ) [8], orthodontic "Ω" wire posts [9], polyethylene fiber posts [10], reversed-oriented metal posts [11], short composite posts [6], glass fiber posts [12], and dentin posts [13].

Dentin posts have shown promising results as these posts can be absorbed as part of the physiological root resorption of primary teeth [14,15], which is considered a special feature of these posts to be the ideal choice for the rehabilitation of severely decayed primary anterior teeth [7]. It is expected that these posts will have similar physical properties of teeth [14].

With the lack of evidence in the medical literature related to the efficiency of dentin posts to restore primary anterior teeth, this study was conducted to determine the ability of these posts to increase the fracture resistance of endodontically treated primary anterior teeth compared with glass fiber posts.

## Materials and methods

In vitro study was planned to compare root fracture resistance between two types of posts (dentine and glass fiber posts) in extracted maxillary primary incisors. The study protocol was approved by the Scientific Research and Postgraduate Board and Ethics Committee of Damascus University, Damascus, Syria (IRB no. UDDS-479-13052019/SRC-2686).

Sample size was determined using the Sample Size Calculation Program (PS Power, version 3.0.43; Nashville, Tennessee: W.D. Dupont and W.D. Plummer). The sample size was calculated using Kathuria et al.'s results, comparing two types of post results in primary teeth [14]. Sample size calculation produced a required sample size of 30 teeth to detect a significant difference (90% power, two-sided 5% significance level). The studied sample was randomly distributed using a lottery, and then they were divided into two equal groups: group I (experimental), which was restored with dentine posts; and group II (control), which was restored with glass fiber posts.

Inclusion criteria were maxillary primary incisors, a single canal, a single root, free of cracks and fractures, straight, no internal absorption, at least two-thirds of the remaining root length, and had not undergone previous endodontic treatment. The sample was collected from the Department of Pediatric Dentistry, Faculty of Dentistry, Damascus University, Damascus, Syria.

The sample was immersed in 10% formalin for 24 minutes and then immersed in saline until the start of the tests. The crowns were cut at a distance of 1 mm above the cemento-enamel junction (CEJ) using diamond discs, and then the root canals were prepared up to #40 H-file (Dentsply Maillefer) with sodium hypochlorite 2.25% irrigation. Root canals were dried using paper points (Ballaigues, Switzerland: Maillefer) and then the canals were filled with Metapex (South Korea: Meta Biomed) based on calcium hydroxide and iodoform. Then, radiographs were taken to make sure that the canals were filled. The post space was prepared using Gates Glidden drills up to size 4 (1.10 mm) in lateral incisors and up to size 5 (1.3 mm) in central incisors, where the extension within the canal was 3 mm below the CEJ.

In the glass fiber posts group, DenMat type (Santa Maria, CA: Den-Mat Corp.) was used with a diameter of 1.2 mm or 1.4 mm to take into account the difference in the incisor canal diameter included in the study, and it was applied as discussed below.

First, the post was wiped with a cotton ball moistened with alcohol and dried without any etching or application of the bond. After ensuring the cleanliness of the dentin walls of the post space, the dentin walls were etched with 37% phosphoric acid (South Korea: Meta Biomed) for 15 seconds and the enamel edges for 30 seconds, then they were washed with water for 10 seconds and dried with gentle air for 10 seconds. Tetric N-Bond (Schaan, Liechtenstein: Ivoclar Vivadent) was applied to all walls and cured for 20 seconds. The post space was filled with dual cure resin cement Variolink II (Schaan, Liechtenstein: Ivoclar Vivadent). Finally, the post was inserted into its space and hardened for 40 seconds, after which the post was cut 3 mm above the CEJ and the crown was constructed using Tetric N-Ceram resin composite (Schaan, Liechtenstein: Ivoclar Vivadent).

**Figure 1:**
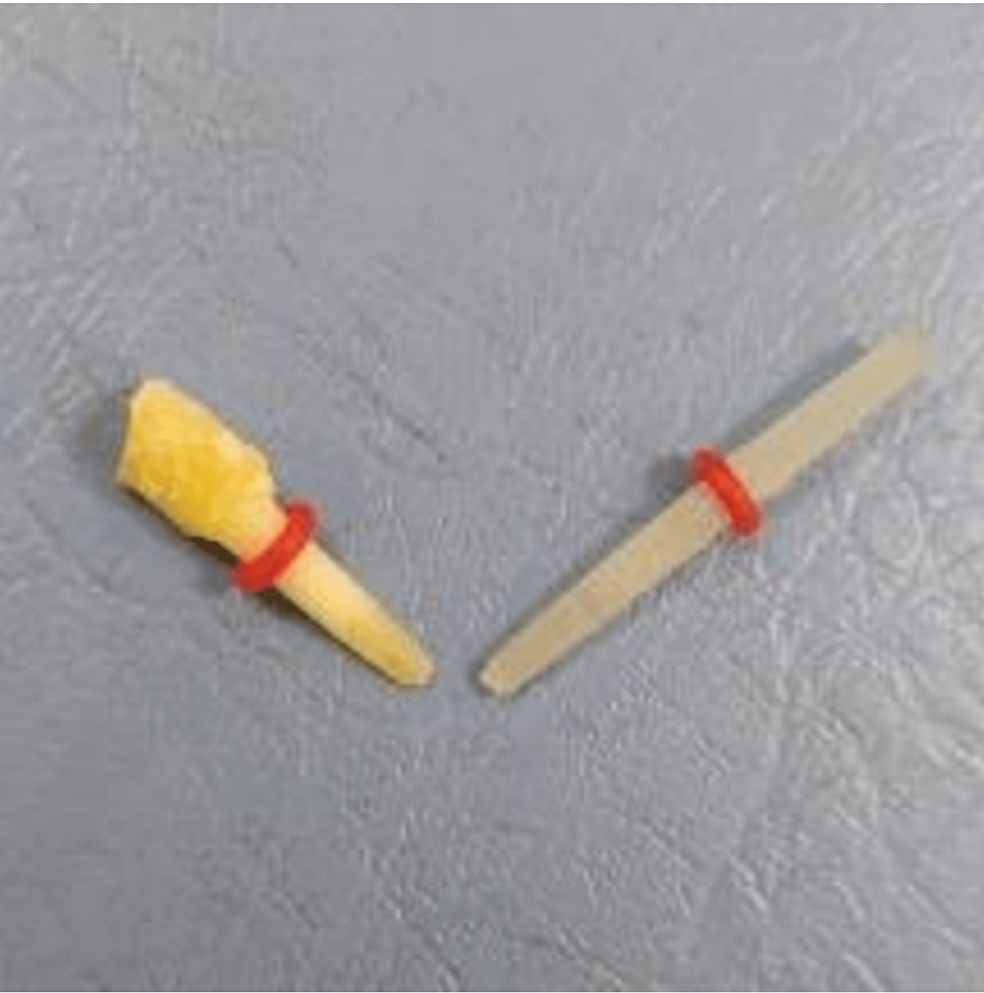
Prepared dentin posts with dimensions that match to glass fiber posts used.

In the dentin posts group, the dentin posts were prepared from extracted, single-root and single-canal permanent teeth (which was confirmed radiologically), where the root was separated from the crown below the CEJ, and the root was then split into two halves, ensuring that the root canal was not present within the prepared post structure. Computer-aided design-computer-aided manufacturing (CAD-CAM) was used to manufacture dentin posts with dimensions of 8 mm in length and 1.2 mm or 1.4 mm in diameter to match the diameters of the glass fiber posts used in this study (Figure 1). The extracted permanent teeth were collected from the Department of Oral and Maxillofacial Surgery, Faculty of Dentistry, Damascus University, Damascus, Syria.

After obtaining the posts, they were immersed in a 10% formalin solution for two weeks according to the protocol approved by the Center for Disease Control (CDC) to sterilize them and were stored in saline until the application, where they were applied according to the previously mentioned fiberglass posts application protocol.

**Figure 2:**
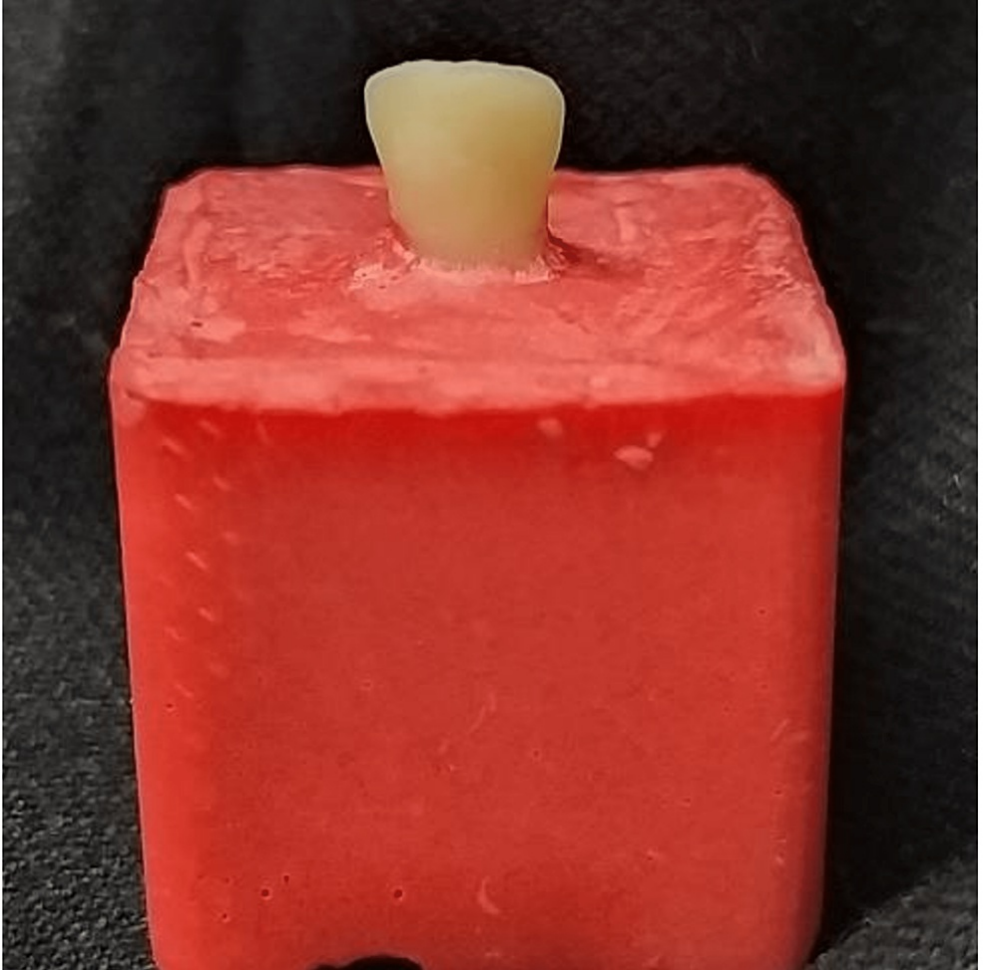
Tooth placed in an acrylic cube.

After building the crowns, the roots were covered with a silicone-based material to simulate periodontal ligament, and then each tooth was placed in an acrylic cube of dimensions (2.5×2.5×2.5 cm) and subjected to 500 thermocycling at 5±1°C to 55±1°C, with a dwell time of 5 seconds (Figure 2).

**Figure 3:**
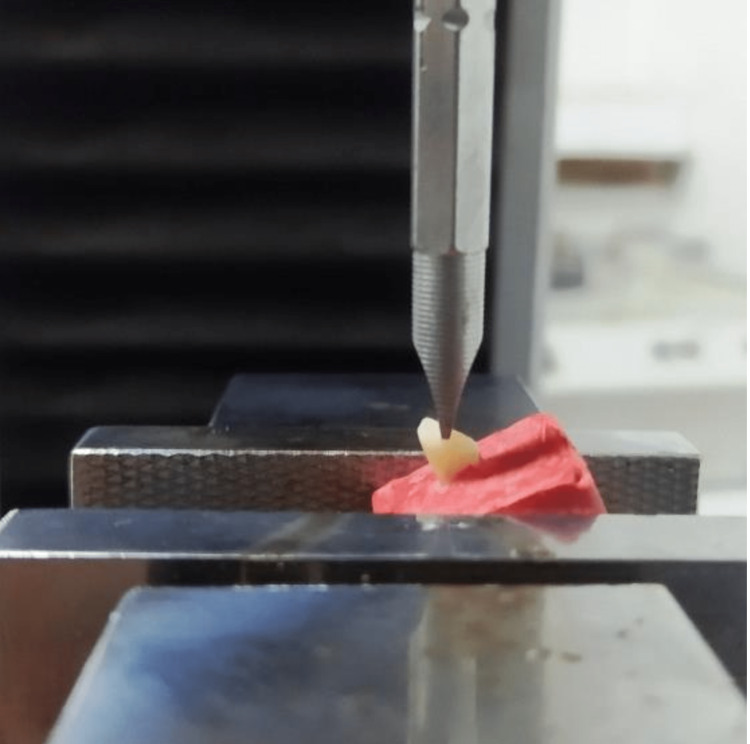
Fracture test application by using a Testometric machine.

The acrylic mold holding the tooth was placed on the base designated for the general mechanical testing device (50kN; Rochdale, England: Testometric Co. Ltd.). A shear force using a conical tip (2.2 mm diameter and speed 1 mm/min) was then applied to the palatal surface of the crown by 45° between the incisal and middle thirds (Figure 3). Force values ​​were recorded in Newtons at the moment of the fracture.

## Results

**Figure 4:**
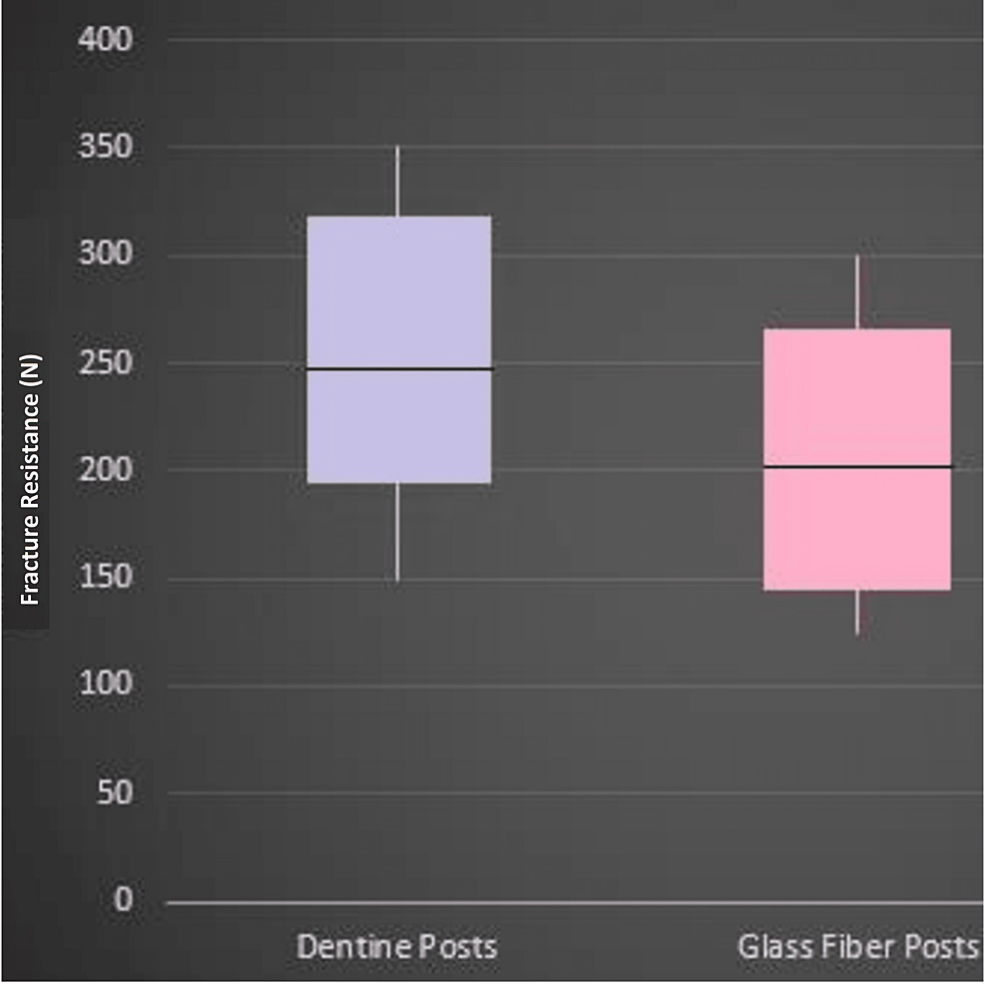
Mean, minimum, and maximum fracture resistance in both study groups. N: Newton

The examined sample consisted of 30 extracted maxillary incisors. Student's t-test was applied to examine the difference in fracture resistance between the two groups of the current study samples, as shown in Table 1 and Figure 4. Table 1 shows that the mean fracture resistance in the glass fiber posts group was 206.3±34.5, while in the dentin posts group, it was 246.3±35.9 with a statistically significant difference (p=0.004) in favor of the dentine posts group.

**Table 1:** Student's t-test results for the differences between the mean fracture resistance between groups.

Group	Means	Standard deviations	Min	Max	p-Value
Glass fiber posts	206.3	34.5	145.0	264.8	0.004
Dentin posts	246.3	35.9	194.7	316.8	

## Discussion

Dental caries and trauma are the most common causes of loss of the coronal primary anterior tooth structure [2]. Rehabilitation of these teeth remains a major challenge for pediatric dentists even with the use of intra-canal retainers, the application of these retainers to primary teeth is more complicated than for permanent teeth, in addition to the requirements for biocompatibility, availability, ease of application, cosmetic aspects, and the ability to withstand chewing forces [16]. The complexity increases due to the physiological absorption process that the roots of primary teeth undergo [17]. Therefore, short posts with lengths not exceeding 3 mm were adopted within the root canal to reconstruct the missing coronal structure [6].

Fracture resistance is one of the most important desirable properties of restorative materials, especially during mastication, and plays an important role in restoration durability [18]. Post application has a significant impact on the final fracture resistance of the restored tooth [19].

This study was done on the maxillary primary incisors, as it is the most affected tooth in early childhood caries, which is in agreement with study done by Gujjar and Indushekar [20]. While other studies included maxillary and mandibular primary canines [13,21].

Metapex was used as a root canal filling which allowed the direct application of posts without the need to apply a spacer such as glass ionomer cement between the canal filler and the posts' adhesive material, this is what is used when filling with zinc oxide and eugenol [21,22].

The dentin posts were prepared from permanent, single-root, and canal teeth that were extracted for orthodontic reasons such as premolars. This agreed with the study done by Ghazawy and Badran, which used the extracted upper and lower premolars for orthodontic reasons [13]. While Kathuria et al. used the extracted maxillary primary canines due to periodontal diseases [14].

The results of this study showed that teeth restored with dentin posts had higher fracture resistance compared to teeth restored with fiberglass posts. This can be attributed to the physical and mechanical properties, in addition to the contribution of these posts to shock absorption and dispersal of their forces, which can be explained by dentin structure [21].

The mean fracture resistance in the dentin posts group was 246.3 N, which is consistent with many studies such as the study of El-Shaabany and El-Baz conducted on the maxillary and mandibular primary anterior teeth [21]. The results of this study are also in agreement with the results of several studies conducted on permanent teeth such as studies done by Ambica et al. and Kathuria et al. [14,23].

While the results of this study differed from the results of the study done by Ghazawy and Badran, where the mean value of the fracture resistance in dentin groups was 145.8 N [13]. The difference may be justified by the method of sterilization of dentin posts before application, where 10% formalin was used for two weeks in the current study, while they relied on sterilization using an autoclave at 121°C for 20 minutes. The difference can also be justified by the diameter of the posts used, which in the current study was 1.2 mm or 1.4 mm. Whereas, in their study, post space was prepared with pluggers, which means that the Gates-Glidden drills were not used to prepare post space. The mean value of the fracture resistance of the glass fiber post group was 206.3 N, which is in agreement with the studies done by Martin et al., Kadkhodaei et al., and Mittal et al. [12,16,17].

The fracture strength was assessed by applying a single 45° load. This is a major limitation of this study because in clinical conditions, chewing forces are more complex, and loads and forces can be directed in different directions.

## Conclusions

The dentin posts used in restoring severely decayed primary maxillary incisors teeth have significantly increased the fracture resistance of these teeth and showed greater fracture resistance than glass fiber posts. Therefore, dentin posts represent a promising alternative to other posts used in the restoration of these teeth.
